# AMPK-Targeting Effects of (−)-Epicatechin Gallate from *Hibiscus sabdariffa* Linne Leaves on Dual Modulation of Hepatic Lipid Accumulation and Glycogen Synthesis in an In Vitro Oleic Acid Model

**DOI:** 10.3390/ijms26157612

**Published:** 2025-08-06

**Authors:** Hui-Hsuan Lin, Pei-Tzu Wu, Yu-Hsuan Liang, Ming-Shih Lee, Jing-Hsien Chen

**Affiliations:** 1 Department of Medical Laboratory and Biotechnology, Chung Shan Medical University, Taichung City 40201, Taiwan; linhh@csmu.edu.tw (H.-H.L.); wptisherry549@gmail.com (P.-T.W.); 2 Clinical Laboratory, Chung Shan Medical University Hospital, Taichung City 40201, Taiwan; 3 Department of Nutrition, Chung Shan Medical University, Taichung City 40201, Taiwan; aliceliang@gmail.com

**Keywords:** lipid accumulation, insulin resistance, *Hibiscus* leaves, (−)-epicatechin gallate, AMPK, oxidative stress, glycogen synthesis

## Abstract

Metabolic dysfunction-associated steatotic liver disease (MASLD) begins with hepatic lipid accumulation and triggers insulin resistance. *Hibiscus* leaf extract exhibits antioxidant and anti-atherosclerotic activities, and is rich in (−)-epicatechin gallate (ECG). Despite ECG’s well-known pharmacological activities and its total antioxidant capacity being stronger than that of other catechins, its regulatory effects on MASLD have not been fully described previously. Therefore, this study attempted to evaluate the anti-MASLD potential of ECG isolated from *Hibiscus* leaves on abnormal lipid and glucose metabolism in hepatocytes. First, oleic acid (OA) was used as an experimental model to induce lipid dysmetabolism in human primary hepatocytes. Treatment with ECG can significantly (*p* < 0.05) reduce the OA-induced cellular lipid accumulation. Nile red staining revealed, compared to the OA group, the inhibition percentages of 29, 61, and 82% at the tested doses of ECG, respectively. The beneficial effects of ECG were associated with the downregulation of SREBPs/HMGCR and upregulation of PPARα/CPT1 through targeting AMPK. Also, ECG at 0.4 µM produced a significant (*p* < 0.01) decrease in oxidative stress by 83%, and a marked (*p* < 0.05) increase in glycogen synthesis by 145% on the OA-exposed hepatocytes with insulin signaling blockade. Mechanistic assays indicated lipid and glucose metabolic homeostasis of ECG might be mediated via regulation of lipogenesis, fatty acid β-oxidation, and insulin resistance, as confirmed by an AMPK inhibitor. These results suggest ECG is a dual modulator of lipid and carbohydrate dysmetabolism in hepatocytes.

## 1. Introduction

Metabolic dysfunction-associated steatotic liver disease (MASLD), previously well-known as non-alcoholic fatty liver disease (NAFLD), is a progressive condition with pathological characteristics ranging from hepatic lipid accumulation to inflammation combined with fibrosis, the stage considered as steatohepatitis [1,2]. Furthermore, cases of MASLD plus steatohepatitis will be further designated as metabolic dysfunction-associated steatohepatitis (MASH) [2,3]. The prevalence of disease progression from MASLD to MASH is estimated at about 12–40% in the general population [3]. The mechanisms underlying the MASH progression have not been thoroughly studied, and untreated or unmanageable hepatic damages cause cell inflammatory injury, death, and extracellular matrix (ECM) deposition in MASLD patients [4]. This damage can manifest in various forms, including cell necrosis or apoptosis and dysfunction. The deposition of excessive ECM is the hallmark of liver fibrosis, leading to gradual liver stiffening [5]. Thus, the inhibition of early-stage MASLD is a potential strategy for clinical treatment of MASH or other metabolic disorders.

Elevating lipid synthesis and/or inhibiting fatty acid (FA) β-oxidation are critical events in modulating lipid accumulation in MASLD [6]. Many studies have found that sterol regulatory element-binding proteins (SREBPs) modulate lipid metabolism [6,7]. Among SREBPs, SREBP-1 modulates the expression of enzymes, acetyl-CoA carboxylase (ACC) and fatty acid synthase (FAS), for lipogenesis involved in triglyceride (TG) synthesis [7]. SREBP-2 acts as a regulator in the transcription of genes associated with cholesterol metabolism, such as low-density lipoprotein (LDL) receptor, 3-hydroxy-3-methylglutaryl-CoA reductase (HMGCR), and SREBP-2 itself [8]. Moreover, peroxisome proliferator-activated receptors (PPARs), categorized as nuclear FA receptors, are highly expressed in the liver, heart, and kidney, and have also been shown to coordinate transcriptional activation of carnitine palmitoyltransferase I (CPT1) for FA β-oxidation [9]. Besides PPARs and CPT1, adenosine monophosphate-activated protein kinase (AMPK), a serine/threonine kinase composing a heterotrimeric protein complex, is regarded as a critical energy sensor in most tissues and controls hepatic lipolysis in response to hormonal and metabolic conditions [10]. In the liver, AMPK activation phosphorylates and then inactivates the rate-limiting enzymes of FA and cholesterol biosynthesis, involving ACC and HMGCR, respectively [11].

Lipid accumulation in the cytoplasm of hepatocytes is a significant cause of insulin resistance, closely related to type 2 diabetes mellitus (T2DM) and metabolic symptoms [12]. The process in insulin resistance is featured by hepatic insulin signaling blockage, leading to an elevated serum level of blood glucose and a lowered hepatic content of glycogen [13,14]. Previous studies have reported that loss function of insulin receptor substrate (IRS) is an important molecular signature of hepatic insulin resistance [14]. IRS is a family protein of docking molecules connecting insulin receptor activation to essential downstream kinase cascades, and its tyrosine (Tyr) phosphorylation leads to insulin signaling activation. By contrast, IRS-1 serine (Ser) phosphorylation results in decreased activities of phosphatidyl inositol 3 kinase (PI3K)/protein kinase B (PKB), contributing to glycogen synthase (GS) inhibition by glycogen synthase kinase-3beta (GSK-3β) triggers a decline in glycogen synthesis in the liver and muscles, and a rise in blood glucose [15].

Recently, FDA had approved Resmetirom (Rezdiffra) which targets thyroid hormone receptor-β for patients with non-cirrhotic MASH, moderate-to-advanced liver fibrosis,, with this approval introducing the first drug for MASLD [16]. Furthermore, dietary intervention, such as specific dietary components, natural products, and herbal medicine, has shown promising therapeutic effects against MASLD and related diseases [17]. *Hibiscus* leaves are an edible and nutritional part of *Hibiscus sabdariffa* Linne (*Malvaceae*; commonly known as Roselle) and are used primarily as leafy vegetables in cooking, soups, and pickle preparations in regions of Africa and Asia [18,19]. Previous studies have found that polyphenolic extract of *Hibiscus* leaves has various biological benefits, and its hypolipidemic [18], anti-atherogenic [19,20,21], antioxidant, and anti-inflammatory [18,22] effects are attributed to its high favonoid content. For the standardization of the extract, further studies have reported (−)-epicatechin gallate (ECG) (16.50%) to be the most abundant substance in polyphenolic extract of *Hibiscus* leaves, followed by ellagic acid (10.33%) [20]. In the literature, a type of active catechin, ECG, is not only an important ingredient of *Hibiscus* leaves, but also a natural dietary antioxidant present in green tea, buckwheat, and grape [21,23]. In vitro and in vivo experimental research indicated that ECG exhibits strong biological capacity in some respects, including antioxidant, anti-inflammatory, antimicrobial, and anti-glycation effects [24,25]. Further study has demonstrated the anti-glycation of ECG is associated with its antioxidant action, and the ECG’s total antioxidant activity was significantly stronger than that of another catechin, epigallocatechin-3-gallate (EGCG) [26]. Studies have demonstrated that ECG could attenuate the abilities of *α*-glucosidase and *α*-amylase related to T2DM [25,27]. According to Wu et al., the opposite effect of ECG on protein glycation might be due to its trapping ability for the critical advanced glycation end products (AGEs) precursor, methylglyoxal (MGO), through the generation of ECG-MGO adducts, further attenuating the AGEs formation [25]. Further research has indicated that ECG was more potent than EGCG in reducing insulin resistance in the palmitate-treated mouse skeletal muscle C2C12 cells [28]. Because little information about anti-MASLD potential and the mechanism of ECG from *Hibiscus* leaves is available, the detailed signaling pathway involved in ECG-mediated beneficial regulation of lipogenesis, fatty acid β-oxidation, and insulin resistance in human primary hepatocytes is explored.

## 2. Results

### 2.1. Noncytotoxic Doses of ECG Abolished the OA-Stimulated Cellular Lipid Accumulation

**Figure 1:**
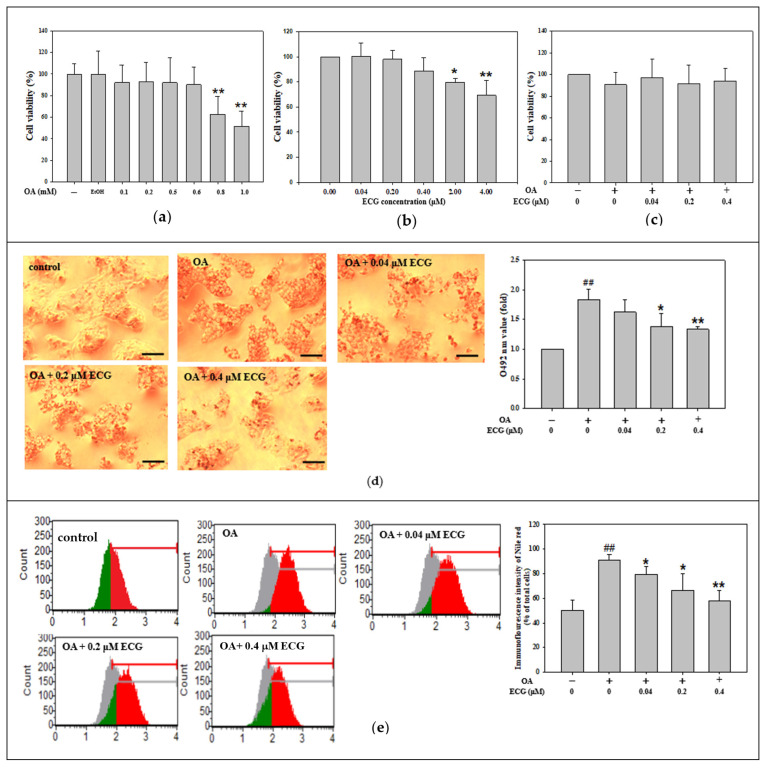
Effect of ECG on cell viability and lipid accumulation in the OA-challenged human primary hepatocytes. Hepatocytes were exposed to increasing concentrations of OA (0.1–1.0 mM) (**a**), ECG (0.04–4.0 µM) (**b**), or co-treated with various concentrations of ECG (0.04, 0.2, and 0.4 μM) with or without OA at 0.6 mM (**c**) for 24 h. EtOH served as a solvent control. Cell viability was assessed using the Muse™ Cell Analyzer. Data are shown as means ± SD (*n* ≥ 3) from three independent replicates. * *p* < 0.05, ** *p* < 0.01 vs. untreated group. (**d**) Lipid accumulation was visualized using oil red O staining and observed under a microscope at 200× magnification; scale bar, 30 μm. Red lipid droplets indicate intracellular fat. For quantification, 1 mL isopropanol was added to dissolve the stain, followed by 5× dilution in ddH_2_O, and absorbance was recorded at 492 nm. (**e**) Nile red staining followed by flow cytometric analysis was used to quantify intracellular lipid content. Data are presented as mean ± SD (*n* ≥ 3) from three independent replicates. ## *p* < 0.01 vs. control; * *p* < 0.05, ** *p* < 0.01 vs. OA group.

The cytotoxicity of OA at different concentrations (0.1–1.0 mM) on human primary hepatocytes for 24 h was evaluated by Muse™ Cell Analyzer (Cytek Biosciences, Fremont, CA, USA) using PI stain. The results indicated that OA had no observable toxic effects on the cells at low doses, 0.1–0.6 mM. However, the cell viability was reduced at doses from 0.8 to 1.0 mM. In addition, the ethanol solvent with the same volume of OA added did not cause the hepatocytes’ cell survival loss (Figure 1a). In this study, the non-toxic dose of 0.6 mM OA was used in later investigations to rule out the impact of cell survival on the observed parameters. Using cell viability experiment, the preliminary concentration screening in a range between 0 and 4 µM was performed to evaluate a 24 h influence of ECG single on hepatocyte growth, indicating there was no toxicity to the cells with doses from 0.04 to 0.4 µM of ECG, while a dose higher than 0.4 µM slightly reduced cell viability (Figure 1b). Furthermore, the quantitative data of Figure 1c confirms that cell survival of hepatocytes was not markedly altered by the treatments with ECG at 0.04–0.4 μM in a 24 h-stimulation of OA.

To determine whether ECG protected hepatocytes from OA-induced lipid accumulation, a set of classical assays, including the oil red O stain and Nile red methods, was utilized to evaluate cellular lipid dysmetabolism. OA increased the amount of oil-loaded cell population nearly 2.0-fold (Figure 1d). Figure 1d indicates that the ECG treatments concentration-dependently reduced the cellular lipid accumulation induced by OA. Next, the Nile red fluorescent staining followed by flow cytometric analysis showed that, compared to the OA-exposed cell group, lower immunofluorescence intensity of Nile red was presented in the cells co-treated with ECG (Figure 1e), which indicated in a similar variation tendency as the data of oil red O stain (Figure 1d).

### 2.2. ECG Inhibited the OA-Induced Increase in Intracellular Lipid Content, Involving Regulation of Lipogenesis and Lipolysis

**Figure 2:**
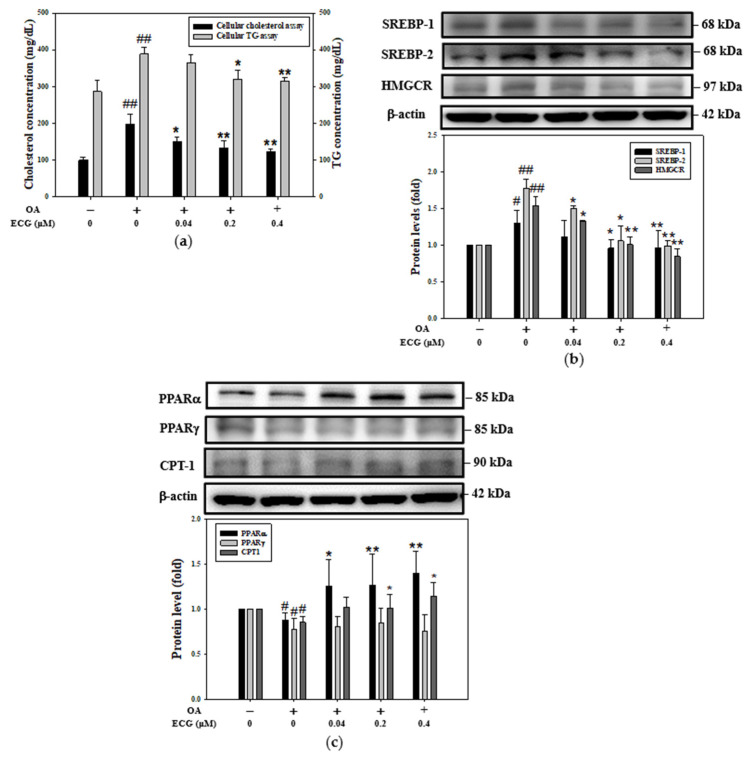
Effect of ECG on lipogenesis and lipolysis in the OA-treated human primary hepatocytes. Hepatocytes were exposed to different concentrations of ECG (0.04, 0.2, and 0.4 μM) with or without OA at 0.6 mM for 24 h. (**a**) Total cellular cholesterol (left axis) and TG (right axis) level were assayed utilizing enzymatic colorimetric assays and presented as mg/dL. The protein levels of lipogenesis-related markers (SREBP-1, SREBP-2, and HMGCR) (**b**) and lipolysis-associated proteins (PPARα, PPARγ, CPT1) (**c**) were determined by Western blotting. β-actin was used as an internal control. Results are shown as mean ± SD (*n* ≥ 3) from three independent replicates. # *p* < 0.05, ## *p* < 0.01 vs. control; * *p* < 0.05, ** *p* < 0.01 vs. OA group.

To determine the improving effect of ECG treatments on cellular cholesterol and TG contents in the OA-exposed hepatocytes, the concentrations of both lipids in cell lysates were measured (Figure 2a). Compared to the control, the cellular total cholesterol and TG contents of hepatocytes were significantly increased by treatment with OA at 0.6 mM. The reduction in the amount of cholesterol (left axis, Figure 2a) was more remarkable than that of TG (right axis, Figure 2a)—it was decreased by about 78% after treatment with ECG at a concentration of 0.4 µM. HMGCR is the rate-determining enzyme involved in cholesterol biosynthesis [6]. To detect whether the ECG decreased lipid content in the OA-challenged hepatocytes was accompanied by changes in cellular cholesterol synthesis, Western blotting was conducted. As shown in Figure 2b, the cellular levels of SREBP-1, SREBP-2, and HMGCR were significantly reduced by ECG treatments compared to the OA-stimulated group, concomitantly with a notable reduction in cholesterol content (left axis, Figure 2a). For the evaluation of lipogenesis and lipolysis, which involve TG metabolism, ACC, FAS, PPARs, and CPT1 are documented as the regulators of OA-induced TG biosynthesis. After a 24 h incubation with OA alone, the cellular levels of PPARα, PPARγ, and CPT1 were reduced (Figure 2c), accompanied by slightly increased levels of ACC and FAS. Notably, ECG treatments corrected the decline in the cellular levels of PPARα and CPT1 in a concentration-dependent manner (Figure 2c).

### 2.3. AMPK Is Essential for the Influence of ECG on the OA-Mediated Lipid Dysmetabolism

**Figure 3:**
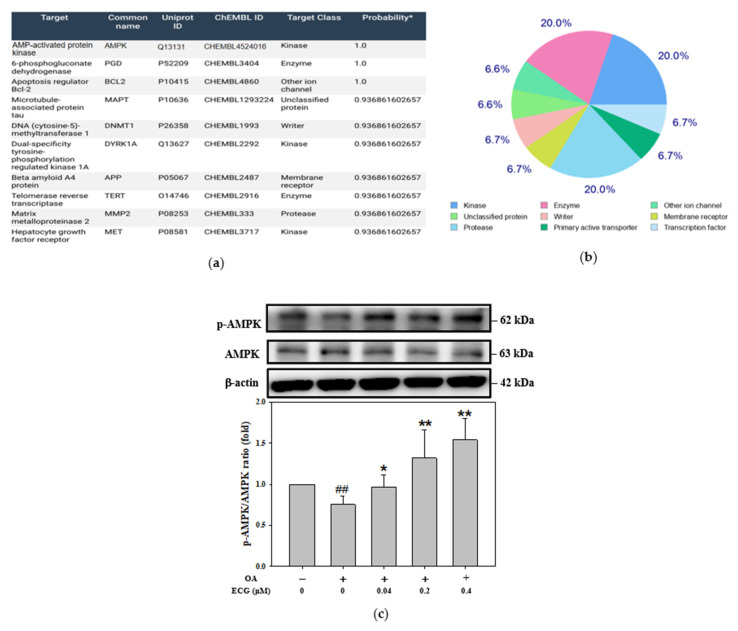
Involvement of AMPK in ECG’s action on the OA-treated human primary hepatocytes. * the estimated value of the most probable targets of bioactive molecules (**a**) The AMPK-targeting (blue marked) of ECG was analyzed utilizing the SwissTargetPrediction. (**b**) The distribution of predicted ECG targets across functional protein classes is shown in a pie chart. (**c**) Western blot analysis was performed to detect p-AMPK, total AMPK, and β-actin, an internal control, after a 24 h treatment of ECG (0.04, 0.2, and 0.4 μM) with or without OA (0.6 mM). Results are shown as mean ± SD (*n* ≥ 3) from three independent replicates. ## *p* < 0.01 vs. control; * *p* < 0.05, ** *p* < 0.01 vs. OA group.

The AMPK-targeting of ECG was evaluated by using the 2019 version of SwissTargetPrediction analytical program (Figure 3a). As shown in Figure 3b, a summary of the predicted target class, presented as a pie chart, proposes that AMPK may participate in the regulatory mechanism of ECG on cellular lipid dysmetabolism upon OA challenge. Similarly, OA downregulated the phosphorylation of AMPK in the cells. Compared to the cells stimulated with OA, the phosphorylated level of AMPK was significantly increased by ECG treatments, while ECG at 0.4 µM was demonstrated to be most potent (Figure 3c).

**Figure 4:**
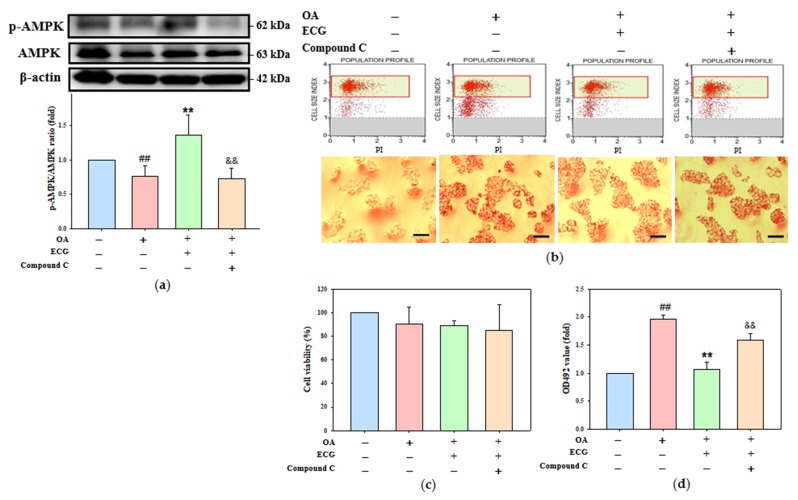
AMPK is essential for the ECG-inhibited lipid accumulation in the OA-challenged human primary hepatocytes. Hepatocytes were pre-treated with compound C (3 µM), then co-treated with ECG (0.4 µM) and OA (0.6 mM) for 24 h. (**a**) The protein levels of p-AMPK and total AMPK were determined by Western blotting. β-actin was used as an internal control. The quantitative data of p-AMPK/AMPK are shown as mean ± SD (*n* ≥ 3) from three independent replicates. (**b**) Cell viability analysis (top) and oil red O staining (bottom) were performed. Images were taken at 200× magnification; scale bar, 30 μm (**c**) Quantitative analysis of cell viability is shown as mean ± SD (*n* ≥ 3) from three independent replicates. (**d**) Lipid staining was quantified by extracting oil red O with isopropanol and reading absorbance at 492 nm. Results are shown as mean ± SD (*n* ≥ 3) from three independent replicates. ## *p* < 0.01 vs. control; ** *p* < 0.01 vs. OA group; && *p* < 0.01 vs. OA + ECG group. (Blue column: control; Red column: OA group; Green column: OA + ECG group; Orange column: OA + ECG + Compound C group).

To explore whether the observed rise in AMPK activation was responsible for the correction of cellular lipid accumulation, which has been indicated to be implicated in the modulation of ECG upon OA challenge, an AMPK inhibition test was performed. Hepatocytes were pre-added to a pharmacological AMPK inhibitor compound C for 30 min [29], and further co-incubated with ECG at 0.4 µM under a 24 h stimulation of OA (0.6 mM). As seen in Figure 4a, the inactivation of AMPK, in the co-incubation of OA plus ECG, remarkably blocked the ECG-enlarged AMPK phosphorylation, without interfering with cell viability (upper panel, Figure 4b,c). The data demonstrated the critical impact of AMPK on the inhibitory potential of ECG toward intracellular lipid accumulation in the OA stimulation (lower panel, Figure 4b–d).

### 2.4. AMPK Is Essential for the Effect of ECG on Lipogenesis and FA β-Oxidation in the OA-Treated Cells

**Figure 5:**
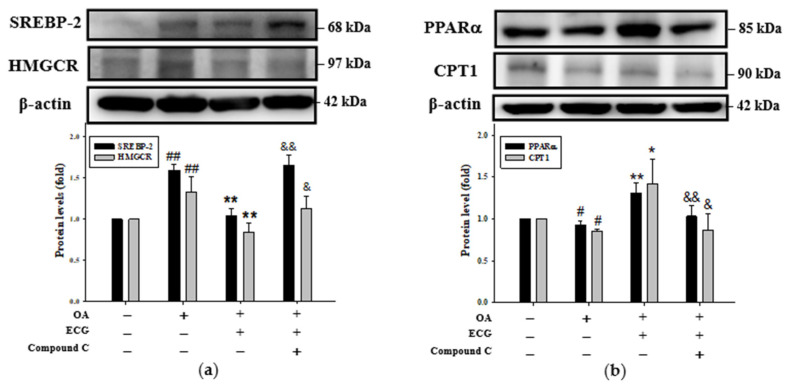
AMPK is essential for ECG-regulated lipogenesis and lipolysis in the OA-treated human primary hepatocytes. Hepatocytes were pre-treated with compound C (3 µM), followed by ECG (0.4 µM) and OA (0.6 mM) treatment for 24 h. Western blotting was used to assess expression of SREBP-2, HMGCR (**a**), PPARα, and CPT1 (**b**). β-actin was used as an internal control. Results are shown as mean ± SD (*n* ≥ 3) from three independent replicates. # *p* < 0.05, ## *p* < 0.01 vs. control; * *p* < 0.05, ** *p* < 0.01 vs. OA group; & *p* < 0.05, && *p* < 0.01 vs. OA + ECG group.

Since the cellular levels of SREBPs and HMGCR were attenuated as the ratio of p-AMPK/AMPK remarkably rose in the group of OA and ECG co-treatment when compared to OA-alone group (Figure 3c), the dependence of ECG-enhanced β-oxidation on the AMPK pathway was evaluated. In the presence of OA, pre-treatment with compound C partially blocked the ECG-downregulated expressions of cholesterol lipogenesis factors SREBP2 and HMGCR (Figure 5a). It should be noted the inhibition of AMPK also abolished the ECG-induced expression of FA β-oxidation factors PPARα and CPT1 (Figure 5b). Taken as a whole, in OA challenge, the inhibition of AMPK impaired the ECG-mediated lipid metabolic homeostasis by interfering with not only lipogenesis but also FA β-oxidation.

### 2.5. AMPK Is Partially Responsible for the Impact of ECG on Glycogen Synthesis and Insulin Signaling in the OA-Treated Cells

**Figure 6:**
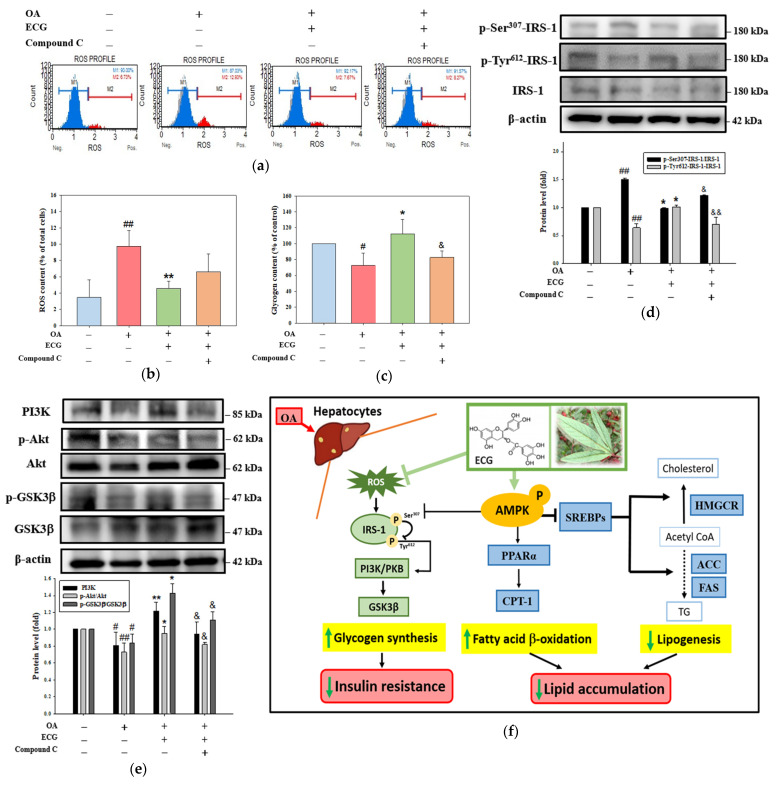
AMPK is essential for the ECG-corrected glycogen synthesis and insulin signaling in the OA-treated human primary hepatocytes. Hepatocytes were pre-treated with compound C (3 µM), followed by ECG (0.4 µM) and OA (0.6 mM) treatment for 24 h. (**a**) The cellular ROS were evaluated using DCFH-DA staining and flow cytometry. (**b**) The percentage of DCF-positive cells was calculated. (**c**) Cellular glycogen levels were measured using ELISA. Results are shown as mean ± SD (*n* ≥ 3) from three independent replicates (Blue column: control; Red column: OA group; Green column: OA + ECG group; Orange column: OA + ECG + Compound C group). Western blotting assessed levels of p-IRS-1 (Ser^307^ and Tyr^612^), IRS-1 (**d**), PI3K, p-PKB, PKB, p-GSK3β, and GSK3β (**e**). β-actin was used as an internal control. Results are shown as mean ± SD (*n* ≥ 3) from three independent replicates. # *p* < 0.05, ## *p* < 0.01 vs. control; * *p* < 0.05, ** *p* < 0.01 vs. OA group; & *p* < 0.05, && *p* < 0.01 vs. OA + ECG group. (**f**) The overview of anti-MASLD effect of ECG from *Hibiscus* leaves (right illustration) on the OA-induced lipid and carbohydrate dysmetabolism in hepatocytes through activating AMPK. By targeting AMPK, ECG functions against the OA’s effects involving the downregulation of hepatic lipogenesis, the upregulation of fatty acid β-oxidation (bold arrows; dashed arrows: the part to further explore), and promotion of p-Tyr-IRS-1/PKB/GSK3β axis-mediated glycogen synthesis (thin arrows), ultimately reducing lipid accumulation and insulin resistance.

To investigate the role of AMPK in the beneficial outcomes of ECG, the ability of compound C to reverse the impact of ECG on oxidative stress-mediated imbalance of carbohydrate metabolism and/or blockade of insulin signaling was further analyzed. Compared to the untreated control, the intercellular levels of reactive oxygen species (ROS) were significantly raised in the OA-exposed cells, contributing to oxidative stress generation. Such a raise was reversed by treatment of ECG, representing its antioxidant action (Figure 6a,b). However, compound C did not exert an inhibitory action on the intercellular ROS content (Figure 6b), suggesting the upstream role of ROS on the AMPK pathway. The results further indicated that compound C neutralized this improvement impact of ECG on the OA-attenuated glycogen content (Figure 6c). Still, there was no change in ROS level (Figure 6b). When cells were exposed to OA alone or OA plus ECG, changes in phosphorylation of Ser and Tyr residues of IRS (Figure 6d) confirmed the inhibition of insulin signaling by AMPK inactivation. The cellular level of PI3K and ratios of p-PKB/PKB and p-GSK3β/GSK3β were also affected by the addition of compound C (Figure 6e). The present study examined the antagonist role of ECG on the OA-caused lipid accumulation and insulin resistance. The results suggest that ECG effectively counteracts the OA-induced lipid accumulation in hepatocytes, particularly in cholesterol biosynthesis. The mechanistic study revealed that AMPK majorly participated in the beneficial effect of ECG on SREBP-2/HMGCR-mediated lipogenesis, and PPARα/CPT1-mediated FA β-oxidation. ECG also functioned against the actions of OA via induction of p-Tyr-IRS-1/PKB/GSK3β pathway, subsequently reducing the insulin resistance by scavenging ROS and targeting AMPK (Figure 6f).

## 3. Discussion

Metabolic syndrome, including obesity and insulin resistance, is related to hepatic lipid accumulation, which is frequently diagnosed as MASLD [2,30]. MASLD is a progressive illness that can be developed into MASH via multiple mechanisms, involving a process from early steatosis to the inflammatory stage, coinciding with diverse degrees of advanced fibrosis [2,3,31]. In previous studies, hepatocytes were usually induced by palmitic acid (PA), OA, or their mixture (1:2) to imitate the influx of overfull free FAs into hepatocytes, leading to steatosis in the liver [32,33]. Moravcová et al. indicated that PA belongs to non-esterified saturated FAs and is toxic to human hepatocyte primary cultures and hepatoma cell lines [34], while OA, an unsaturated FA, is less cytotoxic than PA and weakens PA’s toxicity toward hepatocytes in in vitro MASLD models [35]. OA has also been indicated to be more steatotic than PA and to induce ROS production, contributing to the progression of MASLD to MASH [36]. Thus, to avoid the opposite or differential action of OA between PA on cellular lipid deposition and lipotoxicity, a 24 h incubation with a lower concentration of OA at ≤0.6 mM, exhibiting insignificant toxicity to human primary hepatocytes (Figure 1a), was chosen as a cellular inducer of MASLD in all subsequent experiments in lipid accumulation. As expected, the results found increases in lipid distribution of the oil red O, immunofluorescence intensity of Nile red (Figure 1d,e), amount of cholesterol (Figure 2a), and even blockage of insulin signaling related glycogen storage (Figure 6c–e) were markedly elevated in the OA-stimulated hepatocytes, representing the hepatic dysmetabolism of MASLD model in vitro was successfully identified.

Previous studies have determined that *Hibiscus* leaf polyphenols (HLPs, obtained through a methanol extraction procedure, are rich in polyphenols, including ECG, EA, and others [19,20,21]. In these reports, ECG-enriched HLPs were found to suppress oxidized-LDL (ox-LDL) uptake and foam cell formation, induce cholesterol efflux [19], decrease ox-LDL-induced endothelial cell apoptosis [20], and inhibit TNF-α-stimulated VSMC dysfunction [21]. These findings suggest that HLPs have potential as an anti-atherogenic agent. Furthermore, the studies conclusively indicate that HLPs’ anti-atherosclerotic effects may be provided by the biological activities of these polyphenols. However, Zhen et al. highlight that studies comparing extracts with purified compounds often demonstrate differences in antioxidant and anti-inflammatory potency [22]. Although ECG (Figure 3c) and EA have been shown to modulate AMPK [37], it cannot be ruled out that there are potential synergistic effects of purified ECG and other polyphenols present in the extract, which protect hepatocytes from the stimulation with OA. In the future, to examine the in vitro hepatoprotective effect of HLPs, which are primarily caused by ECG, the converted dose of ECG to that of the extract at 0.5 and 1.0 μg/mL should be used. Similar results of cell viability, lipid accumulation assay, and ROS and glycogen synthesis assay are expected to be found in the extract-treated human primary hepatocytes in the presence of OA.

Many functional food ingredients and natural products, such as anthocyanins [38], catechins, or plant extracts [39,40] individually exert modulatory abilities on lipid or carbohydrate metabolism. Recently, dual regulation of natural products on lipid or glucose metabolic homeostasis has been considered more effective in anti-MASLD. Besides *Hibiscus* leaves, ECG is a key ingredient of green tea, showing a lot of biological potential due to its antioxidant, anti-inflammatory, and anti-glycation activities [24,25,26,27,28]. In previous studies, green tea was proved to contain various antioxidant catechins, such as EGCG and ECG, which have potent counteracting effects on LDL oxidation in vitro and ex vivo in humans [24]. Furthermore, ECG-enriched extract has also been noted for its potent antioxidant activity, and has been explored for the development of anti-atherosclerotic agents [20,24]. Unlike the higher doses of ECG (50 and 100 µM) used to exhibit anti-tumorigenic activity [23], ECG at 0.1 μM demonstrated the ability to trap the key AGE precursor, MGO, within 24 hours [25]. After prolonged incubation with PA (48 h), ECG treatment at 1–20 μM was able to restore IRS-1 expression, enhance PKB phosphorylation, and improve glucose uptake in mouse skeletal muscle C2C12 cells [28]. ThAe comparison of antioxidant and metabolic effects of ECG from previous studies and this study is provided in Table S1. Among ECG’s pharmacological activities, the regulatory effects on MASLD, especially in lipid metabolism, has not been fully described previously. However, it has been reported that ECG could potentiate doxorubicin cardiotoxicity to H9C2 cardiomyocytes through activating AMPK [41], showing the potential of ECG on targeting AMPK, as demonstrated by the SwissTargetPrediction analytical program (Figure 3a,b). In the present study, using a well-established model of hepatocytes exposed to OA, to our knowledge, this is the first report indicating the anti-MASLD effect of ECG from *Hibiscus* leaves not only on lipid accumulation but also insulin resistance.

Certain natural products or dietary antioxidants have been further shown to have differential outcomes in different cell types under differing doses [42]. There is still a debate on whether natural products, such as resveratrol, can inhibit cancer cell viability by activating apoptosis, keeping cells from oxidative harm, and enabling normal cell growth [43,44]. In agreement with these past reports, the results showed that ECG at higher dosage suppressed cell proliferation (50 μM) in human colorectal carcinoma HCT-116 cells [23], while, on the other hand, its lower doses (0.2–0.4 μM) exhibited an ability to improve MASLD features in hepatocytes under OA stimulation. Hence, the findings provide evidence supporting the multi-functionality of ECG on HCT-116 cells and hepatocytes in high and low doses, and may reflect differences in experimental design, specifically in cell type-specific responses. In addition, ECG and EGCG are the primary polyphenols in green tea, accounting for about 50–80% of the catechin content [45]. In fact, the main differences between ECG and EGCG lie in their chemical structure, plant source, and biological activity [26,46], supporting the idea that ECG possesses antioxidant activity and exhibits in vitro hepatoprotective and insulin resistance-improving effects comparable to those of EGCG (Table S2). Generally, ECG and EGCG were more effective than other catechins, epicatechin (EC) and epigallocatechin (EGC), in 2,2-diphenyl-1-picrylhydrazyl (DPPH) radical scavenging ability [26] and modulating insulin-stimulated mitogenic signaling [47]. Wang et al. indicated that using 2,2′-azino-bis-(3-ethylbenzothiazoline-6-sulfonic acid) (ABTS^+·^) radical scavenging and ferric reducing antioxidant power (FRAP) methods, the catechins’ antioxidant ability was demonstrated be in the following order—ECG ≈ EGCG > EGC > EC—and the ECG’s total antioxidant capacity was significantly stronger than EGCG’s [26]. Much of the evidence shows that EGCG might be related to benefits in MASLD therapy, which may be due to the modulation of energy metabolism, anti-inflammatory, antioxidant, anti-fibrosis, and other pharmacological mechanisms of EGCG [45]. In the liver of MASLD mice, EGCG concentration-dependently increased insulin sensitivity and secretion, as well as upregulated enzyme activity and protein expression of insulin-degrading enzyme [48]. While the focus has been on EGCG previously, the ECG is also closely implicated in the potential against MASLD. In the future, in vivo experiments need to further investigate ECG’s beneficial effects on moderate lipid and glucose metabolic homeostasis.

The hypolipidemic mechanisms of ECG indicated a low level of cholesterol synthesis (SREBP-2 and HMGCR), and high level of FA β-oxidation (PPARα and CPT1) in hepatocytes under OA stimulation (Figure 2b,c). As depicted in Figure 2a, ECG significantly reduces cholesterol levels by 78% via the SREBP-2/HMGCR pathway, yet its effect on TG is limited to a 30% reduction via PPARα/CPT1, indicating that the observed disparity in ECG’s impact has a greater effect on cholesterol synthesis than on FA oxidation. Chen et al. suggested that EGCG exhibits a higher affinity for SREBPs than for PPARs [45]. The so-called AMPK hypothesis was later proposed by Yang et al. [49]. For example, EGCG has been shown to exert anti-obesity effects through AMPK activation, which triggers the downregulation of SREBP transcription factors and upregulates the expression of two lipogenic enzymes: HMGCR and FASN [45]. Our study is consistent with previous findings that support the involvement of AMPK in ECG’s regulation of lipid metabolic homeostasis. Specifically, we confirmed that ECG influences SREBP-2/HMGCR-mediated cholesterol synthesis and PPARα/CPT1-mediated fatty acid oxidation in hepatocytes, as demonstrated using the pharmacological AMPK inhibitor compound C (Figure 4 and Figure 5). These results suggest that the selectivity of ECG may involve distinct downstream signaling cascades initiated by AMPK. The activation of AMPK modulates the expressions of several downstream factors related to lipid metabolism [38]. In current studies, among the AMPK downstream factors, HMGCR and PPARα have been well recognized. Additionally, Gan et al. assert that in MASLD, signal transductions such as mammalian target of rapamycin (mTOR)-involved lipid synthesis and nuclear factor kappa-light-chain enhancer of activated B cells (NF-κB)-mediated inflammation modulate ACC/FAS [48]. Additional study has also demonstrated that the activation of AMPK blocked the ROS/NF-κB pathway, leading to the attenuation of endotoxemia-induced hepatic injury [50]. In view of this, the increases in total and phosphorylated levels of NF-κB were further observed in the OA-treated human primary hepatocytes, but they were significantly inhibited by ECG interventions. Also, this effect was avoided by Compound C (Figure S1), suggesting the NF-κB inflammatory signaling pathway might explain the residual effects observed after AMPK inhibition.

On the other hand, we also propose that ECG-stimulated activation of AMPK plays a protective role in glycogen synthesis. To directly determine whether AMPK activation contributes to the protective effect of ECG, compound C was pre-treated in hepatocytes to inhibit AMPK phosphorylation (Figure 6c–e). Unexpectedly, compound C-mediated AMPK inhibition only partially hindered the protective action of ECG, suggesting that, besides the AMPK-dependent mechanism, ECG protected hepatocytes against insulin resistance via other targets or regulators. It is widely accepted that ROS can enhance the impairment of the IRS-1/PKB/GSK3β signaling [38,51] and promote the phosphorylation of IRS at Ser residue, which is associated with insulin resistance. Meanwhile, as shown in Figure 6a,b, the treatment with OA could induce the production of intracellular ROS, which might influence the blockage of insulin signaling through stimulating the Ser^307^-phosphorylation of IRS-1 (Figure 6d). In this regard, ECG weakens ROS production against the OA stimulation (Figure 6c), and it might mainly lead to glycogen synthesis and metabolic homeostasis by ECG’s antioxidant ability. A recent meta-analysis confirmed that one of the anti-MASLD effects of EGCG is related to enhanced antioxidant activity, including the activation of nuclear factor erythroid 2–related factor 2 (Nrf2) signaling [52]. Further research is needed to investigate the rationale for Nrf2 or other antioxidant pathways in the ECG’s reduction in ROS, determining whether ECG’s antioxidant action is direct (free radical neutralization) or indirect (Nrf2 activation or increased glutathione).

Currently, the findings provide novel insights into the mechanistic role of ECG in modulating hepatic metabolic homeostasis via AMPK signaling. Nevertheless, some limitations remain: (i) Although an OA model is a well-established in vitro approach for exploring molecular mechanisms, it is too simplistic to reveal all the features of MASLD/MASH in humans. MASLD and MASH are complex conditions in which many factors contribute to pathogenesis. For instance, the inflammatory and oxidative state generated by infiltrating macrophages in the liver of patients with MASLD/MASH is missing in this in vitro model. (ii) Also, since it is now obvious that within any fatty acid class, different members have different actions and effects, the effects of other physiologically relevant fatty acids were further needed to test in vivo. (iii) Another limitation of the present study is the lack of an experimental system that reflects the situation more accurately by comparing the concentrations and efficacy of ECG and polyphenolic extract of *Hibiscus* leaves. ECG is the major component (16.5%) of the extract, but it remains unclear whether this concentration of polyphenolic extract of *Hibiscus* leaves, that is equivalent to that of ECG used in this study, is sufficient to exhibit the observed effects on anti-MASLD. (iv) It also remains unclear whether the observed effects are solely attributable to ECG or result from its potential synergistic effects with other polyphenols (such as EA) present in the extract. (v) Moreover, it would be interesting to evaluate whether OA-triggered pathways such as NF-κB (inflammation) and mTOR (lipid synthesis), which modulate ACC/FAS, are influenced by ECG, and to determine whether ECG’s antioxidant action is direct or mediated through upstream regulatory mechanisms.

## 4. Materials and Methods

### 4.1. Extraction, Identification, and Flavonoid Compound Isolation of Polyphenolic Extract of Hibiscus Leaves

Dried *Hibiscus* leaves were gained from Taitung City, Taitung County, Taiwan, ROC, and their polyphenolic extract was prepared and identified as described by Chen et al. [20]. In accordance with the retention time of high-performance liquid chromatography (HPLC) system applying a Vectra 436/33N system (Hewlett-Packard, Palo Alto, CA, USA) coupled with a diode array detector, a single peak at 345 nm, i.e., the characteristic absorption wavelength of ECG, was collected as the ECG-enriched fraction. A pale solid was precipitated from the fraction to obtain compound **1** (ECG, 413 mg), and then purified after filtration and re-crystallization from methanol [19].

### 4.2. Cell Culture, Treatment, and Viability Assay

Human primary hepatocytes (HMCPIS, Lot Hu8200) were purchased from Thermo Fisher Scientific (Waltham, MA, USA) and cultured as recommended by the supplier. In brief, the cells were thawed in cryopreserved hepatocyte recovery medium, centrifuged, re-suspended in plating medium composed hepatocyte plating supplement pack in William’s E medium, and maintained on collagen-coated 12-well plates at 2.5 × 10^5^ cells. The cells were rinsed twice, and the medium was replaced with fresh incubation medium after a 4 h incubation. The media mentioned above were obtained from Thermo Fisher Scientific. Before treatment, human primary hepatocytes were seeded at a density of 5 × 10^5^ onto 6-well plates for 24 h, and the oleic acid (OA)/bovine serum albumin (BSA), complex solution was prepared according to the previously studied protocol [53]. OA was dissolved in ethanol (EtOH) as a stock solution of OA at 1 M, and then diluted in medium mixed with 10 µL/mL of BSA to achieve the desired final doses. Through a 0.22 µm pore membrane filter, the OA/BSA complex solution was sterile-filtered and further stored at −20 °C.

In a cell model of MASLD, the hepatocytes at 80% confluence were maintained in serum-free medium for 24 h, and exposed to OA at 0.1–1.0 mM or ECG (0.04, 0.2, and 0.4 µM) from Sigma-Aldrich (St. Louis, MO, USA) in the presence or absence of OA at the indicated dose for another 24 h. Human primary hepatocytes’ viability was measured using the Muse™ Cell Analyzer (Cytek Biosciences, Fremont, CA, USA). Briefly, the treated cells were harvested after a 24 h stimulation. The harvested cells were centrifuged at 1000 rpm for 10 min, rinsed once with ice-cold phosphate-buffered saline (PBS), and then placed in 500 µL of PBS containing 10 μL of propidium iodide (PI, 10 μg/mL) for 5 min in the dark, at room temperature. The above chemical agents were obtained from Sigma-Aldrich, Sigma Chemical Co., St. Louis, MO, USA. Furthermore, the samples were analyzed according to the manufacturer’s instructions.

### 4.3. Lipid Accumulation Assay

Oil red O stain. The cellular lipid accumulation was analyzed by oil red O stain, and then quantified by measuring the absorbance at 492 nm utilizing the modified method of Chen et al. [19]. Hepatocytes were exposed to OA (0.6 mM) in the presence or absence of ECG for 24 h. The treated cells were rinsed once with 1× sterilized PBS, followed by paraformaldehyde treatment at 25 °C. After a 30 min fixation, the neutral lipids were then stained with 0.5% oil red O stain in isopropanol for another 60 min. Using microscopy, the stained lipids were evaluated for morphology, and then performed spectrophotometrically after isopropanol extraction.

Nile red stain. Nile red stain was assayed as described [37]. The treated cells were rinsed twice with PBS, fixed with 4% formaldehyde in PBS for 30 min, and further stained with 1 μg/mL Nile red for 40 min at 25 °C. Subsequently, the lipid distribution in the stained cells was detected by Muse™ Cell Analyzer.

### 4.4. Cellular Lipid Content Analysis

According to the procedure, cellular layers were extracted in hexane–isopropanol (3:2, *v*/*v*) [54]. After 1000× *g* centrifugation for 15 min, the lower clear organic phase of the extracted solution was transferred into a glass tube and then lyophilized. Subsequently, the lyophilized powder was dissolved in isopropanol as the cellular lipid extract and stored at −20 °C. The cellular cholesterol and TG contents were analyzed by enzymatic colorimetric methods utilizing commercial kits (Human, Wiesbaden, Germany).

### 4.5. Western Blotting

Protein expression was evaluated utilizing the Western blot analysis described in the previously reported study [55]. In brief, total protein was gained from the harvested cells reacted with radioimmunoprecipitation assay (RIPA) buffer, and the quantification of protein concentration was then performed using the bicinchoninic acid (BCA) protein kit (Thermo Scientific, Rockford, IL, USA). An equal amount of protein (about 50 μg) was electrophoresed through 8–12% SDS-PAGE gels and separated onto nitrocellulose papers (Millipore, Bedford, MA, USA), which were immunoblotted with the primary antibodies against SREBP-1 (sc-13551), SREBP-2 (sc-13552), HMGCR (sc-271595), PPARα (sc-398394), PPARγ (sc-7273), CPT1 (sc-514555), AMPK (sc-74461), IRS-1 (sc-560), PI3K (sc-1637), p-PKB (sc-7985), PKB (sc-8312), p-GSK3β (sc-11757), and GSK3β (sc-9166), purchased from Santa Cruz Biotechnology (Santa Cruz, CA, USA), p-AMPK (2535), p-IRS-1-Ser307 (2381), and p-IRS-1-Tyr612 (3203) from Cell Signaling Technology (Danvers, MA, USA), and anti-β-actin (A5441), obtained from Sigma-Aldrich (St. Louis, MO, USA) at 4 °C overnight after blocking to reduce the background by nonfat dried milk diluted in Tris buffered saline (TBS) for 30 min. Subsequently, the blots were washed and reacted with suitable secondary antibodies for 2 h at 25 °C. Using a LAS-4000 Luminescent Image Analyzer (Fujifilm Corporation, Tokyo, Japan), the signals of protein bands were detected using enhanced chemiluminescence (ECL).

### 4.6. SwissTarget Prediction Analysis

SwissTargetPrediction www.swisstargetprediction.ch (accessed on 22 May 2025) was used to assess and predict the molecular targets of ECG [56].

### 4.7. AMPK Inhibition Test

The hepatocytes were cultured in 6-well plates (5 × 10^5^ cells/well) for 24 h, and pre-treated with AMPK inhibitor, compound C (3 μM) [29], from Sigma Chemical Co. (St. Louis, MO, USA) for 30 min before the OA (0.6 mM) with or without ECG (0.4 µM) for another 24 h. Subsequently, cell viability, oil red O stain, and Western blotting were carried out as described above.

### 4.8. Cellular ROS Measurement

The cellular ROS were assayed by a Muse^®^ Oxidative Stress Kit (Luminex, Austin, TX, USA). After treatments, the cells were harvested and then stained with 2 µM of dichlorofluorescein diacetate (DCFH-DA) for 20 min in the dark. The intensity of fluorescent DCF was immediately measured using the Muse™ Cell Analyzer. The levels of each group were presented relative to the fluorescence intensity of intracellular ROS of the control.

### 4.9. Glycogen Synthesis Assay

After various treatments for 24 h, the cellular glycogen contents of the treated cells were determined using the EnzyChrom^TM^ Glycogen Assay Kit (Human, Wiesbaden, Germany). The absorbance at 620 nm of glycogen levels was analyzed with a microplate spectrophotometer (Thermo Fisher Scientific, Waltham, MA, USA) [57].

### 4.10. Statistical Analysis

All data are represented as means ± standard deviation (SD). A significant difference in statistical assay was performed by one-way analysis of variance (ANOVA) and established at *p* < 0.05.

## 5. Conclusions

Taken together, this study first demonstrated that ECG isolated from *Hibiscus* leaves is effective in regulating SREBPs, PPARs, and their individual downstream factors HMGCR and CPT1, as well as IRS-1/PI3K/PKB/GSK3β signaling through targeting AMPK, at least in part, leading to a benefit in the hepatic lipid and carbohydrate homeostasis upon OA challenge. The results of this study revealed that ECG plays a significant role in alleviating lipid accumulation and regulating carbohydrate metabolism, and might hold value in the therapy of MASLD.

## Data Availability

The original contributions presented in the study are included in the article, further inquiries can be directed to the corresponding authors.

## Supplementary Materials

The following supporting information can be downloaded at https://www.mdpi.com/article/10.3390/ijms26157612/s1.

## Abbreviations

MASLD	metabolic dysfunction-associated steatotic liver disease
MASH	metabolic dysfunction-associated steatohepatitis
ECM	extracellular matrix
FA	fatty acid
SREBPs	sterol regulatory element-binding proteins
ACC	acetyl-CoA carboxylase
FAS	fatty acid synthase
TG	triglyceride
HMGCR	3-hydroxy-3-methylglutaryl-CoA reductase
LDL	low-density lipoprotein
PPARs	peroxisome proliferator-activated receptors
CPT1	carnitine palmitoyltransferase I
AMPK	adenosine monophosphate-activated protein kinase
PI3K	phosphatidyl inositol 3 kinase
PKB	protein kinase B
GSK-3β	glycogen synthase kinase-3beta
ECG	(−)-epicatechin gallate
SDS-PAGE	sodium dodecyl sulfate-polyacrylamide gel electrophoresis
TBS	tris-buffered saline
ECL	enhanced chemiluminescence
