# Supplementary material for: AMPK-Targeting Effects of (−)-Epicatechin Gallate from Hibiscus sabdariffa Linne Leaves on Dual Modulation of Hepatic Lipid Accumulation and Glycogen Synthesis in an In Vitro Oleic Acid Model
Source: Int J Mol Sci. 2025 Aug 6;26(15):7612. doi: 10.3390/ijms26157612 (PMC12347588; doi:10.3390/ijms26157612)
Supplement: Supplementary file 1 [file ijms-26-07612-s001.zip › ijms-3737391-supplementary.pdf]

Table S1. The comparison of antioxidant and metabolic impact of ECG in previous studies versus this study

Effect	Model	Dosage & time	Outcome	Reference
**Anti-atherosclerosis**	an atherogenic diet-fed C57BL/6J, apoprotein (apo) E-deficient **mice**	Drinking water supplemented with green tea extract (0.8 g/L), consisted of 0.5 g ECG/100 g extract, for 14 wk	1. Plasma lipid peroxides were reduced in the tea group at wk 8.<br>2. Atheromatous areas in the aorta from the arch to the femoral bifurcation and aortic weights were both significantly attenuated in the tea group.<br>3. Aortic cholesterol and triglyceride contents were lower in the tea group.	Miura, Y. et al. *J. Nutr.* **2001**, *131*, 27–32. [24]
**Anti-tumorigenic**	HCT-116 and SW480 human **colorectal cells**	0.1, 1, 10, 50, and 100 μM for 24 h	1. ECG induces activating transcription factor 3 (ATF3), which is involved in pro-apoptosis in HCT-116 cells.<br>2. 50, and 100 μM ECG produces oxidative stress in the cell culture media, followed by the increased expression of early growth response gene-1 (EGR-1), a tumor suppressor protein, inducting ATF3 protein to protect cells from the extracellular stress signal	Cho, K.N. et al. *Eur. J. Cancer* **2007**, *43*, 2404–2412. [23]
**Endothelial-protective**	**Human umbilical vein endothelial cells** (HUVECs) exposed to ox-LDL in vitro	0.4, 2, 4, and 20 μM for 24 h	1. ECG showed potential in reducing ox-LDL-dependent HUVECs apoptosis.<br>2. the ox-LDL-induced formation of acidic vesicular organelles and upregulation of the autophagy-related genes were increased by ECG<br>3. ECG-enriched extracts upregulates the autophagic pathway, which in turn led to reduce ox-LDL-induced HUVECs apoptosis	Chen, J.H.; *Eur. J. Nutr.* **2017**, *56*, 1963–1981. [20]
**Anti-glycation**	An ***in vitro*** Bovine serum albumin (BSA) -fructose model	8.9, 15.5, 22.1, 33.0, 44.2 and 88.5 μg/mL for 24 h	1. ECG suppressed the carbonylation and the formation of amyloid cross-β structures of BSA as well as the generation of AGEs in this model.<br>2. ECG at 44.2 μg/mL (equivalent to 0.1 μM) showed a trapping efficiency for the important AGEs precursor MGO within 24 h.	Wu, X. et al. *Food Res. Int.* **2019**, *122*, 230–240. [25]
**Anti-insulin resistance**	12-O-tetradecanoylphorbol 13-acetate (TPA), a protein kinase C (PKC) activator, and palmitate to induce insulin resistance in C2C12 mouse **skeletal muscle cells**	20 μM for 10-60 min, and 1-20 μM for 24-72 h	1. After 5 h of palmitate incubation, ECG can suppress IRS-1 Ser307 phosphorylation and significantly promote Akt, ERK1/2, p38 MAPK, and AMPK activation.<br>2. With a longer incubation with palmitate, treatment with ECG could reverse IRS-1 expression and Akt phosphorylation, and improve glucose uptake in C2C12 cells<br>3. ECG was more effective than EGCG in attenuating insulin resistance.	Deng, Y.T. et al. *J. Agric. Food Chem.* **2012**, *60*, 1059–1066. [28]
**Antioxidant**	1,1-Diphenyl-2-picrylhydrazyl (DPPH), 2,20-Azinobis (3-ethylbenzothiazoline-6-sulfonic acid, $ABTS^{+}$) and total antioxidant capacity assay in **non-cellular system**	100, 250, and 500 μM	1. In terms of the scavenging $ABTS^{+}$ free radical, ECG was significantly stronger than (-)-epicatechin (EC), (-)-gallocatechin (GC), and (-)-catechin gallate (CG), respectively.<br>2. With regard to the scavenging DPPH free radicals, ECG were significantly stronger than GC and CG. There were no significant differences between EGCG and ECG on the DPPH free radical scavenging activity<br>3. The total antioxidant capacity of ECG was significantly stronger than Trolox	Wang, W. et al. *Foods* **2023**, *12*, 4207. [26]
**Anti-MASLD**	**Human primary hepatocytes**	0.04, 0.2, and 0.4 μM for 24 h	1. 0.4 μM ECG reduced cellular lipid accumulation and dysmetabolism.<br>2. The beneficial effect of ECG was associated with downregulation of SREBPs/ HMGCR and upregulation of PPARα/ CPT1 through activating AMPK (Thr172 phosphorylation).<br>3. 0.4 μM ECG exhibited oxidative stress clearance and glycogen synthesis-promoting effects on the OA-treated cells with insulin signaling blockade, including IRS-1 Ser307 phosphorylation ↓ , Tyr612 phosphorylation ↑ and Akt/GSK3β phosphorylation ↑ .	Lin, H.H. et al. this study, **2025.**

Table S2. The comparison of (−)-epicatechin gallate (ECG) *vs.* (-)-epigallocatechin-3-gallate (EGCG)

Catechins / Compared item	(−)-Epicatechin gallate (ECG)	(-)-Epigallocatechin-3-gallate (EGCG)
**Chemical structure**[1]	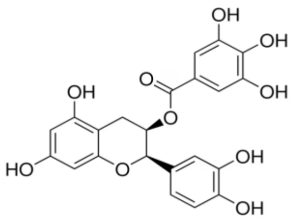	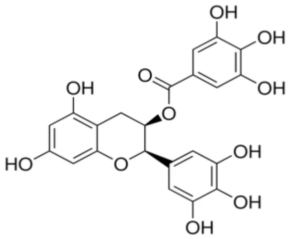
**Molecular formula**	$C_{22}H_{18}O_{10}$	$C_{22}H_{18}O_{11}$
**Molecular weight (g/mol)**	442.37	458.37
**Plant source**[2]	Green tea (0.5%), buckwheat, grape, and *Hibiscus sabdariffa* leaf	Green tea (approximately 50-80%)
**Biological function**		
**- Antioxidant activity**[3]		
DPPH radical scavenging assay	DPPH scavenging rate: 75% (≅)	DPPH scavenging rate: 75%
$ABTS^{+}$ radical scavenging assay	$ABTS^{+}$scavenging rate: 60% (<)	$ABTS^{+}$scavenging rate: 65%
Total antioxidant capacity assay with the FRAP method	8.41 ± 0.17 mM $FeSO_4$ equivalents (>)	7.61 ± 0.1 mM $FeSO_4$ equivalents
**- Hepatoprotective effect**	1. In oleic acid (OA)-induced human primary hepatocytes, 0.4 μM ECG reduced cellular lipid accumulation and dysmetabolism by about 60%-80%.[4]<br>2. The beneficial effect of ECG was associated with downregulation of SREBPs/ HMGCR and upregulation of PPARα/CPT1 through activating AMPK (Thr172 phosphorylation).[4]	1. In vivo models of MASLD, not in human MASLD, EGCG promoted lipid and glucose metabolism, anti-lipid peroxidation and anti-inflammation activities, anti-fibrosis, and anti-MASLD related tumor.[5,6]<br>2. 10 μM EGCG inhibited hepatic lipogenesis by 65% by increasing expressions of p-AMPKα (Thr172) and p-ACC (Ser79) in high-glucose treated HepG2 cells.[5,7]
**- Insulin resistance-improvement effect**	1. ECG at 44.2 μg/mL (equivalent to 0.1 μM) showed a trapping efficiency of 81.6% for the important AGEs precursor MGO within 24 h.[2]<br>2. With a longer incubation with palmitate, treatment with ECG could reverse IRS-1 expression and Akt phosphorylation, and improve glucose uptake in skeletal muscle C2C12 cells.[8]<br>3. In oleic acid (OA)-induced human primary hepatocytes, 0.4 μM ECG exhibited oxidative stress clearance (83%) and glycogen synthesis-promoting effects (1.3-fold) on the OA-treated cells with insulin signaling blockade, including IRS-1 Ser307 phosphorylation↓, Tyr612 phosphorylation↑ and Akt/GSK3β phosphorylation↑.[4]	1. EGCG (10, 20 and 40 mg/kg) dose-dependently increased insulin sensitivity, secretion, and upregulated insulin-degrading enzyme protein expression and enzyme activity in the liver of MASLD mice.[9]<br>2. In vitro, glycogen synthesis (41/53% with 0.1/1 μM EGCG; 2-fold with 10 μM EGCG)↑, and Tyr phosphorylation of IRS-1 and Akt Ser473 phosphorylation↑.[5,10]
**Reference**	[1] Miura, Y. et al. *J. Nutr.* **2001**, *131*, 27–32 [24]; [2] Wu, X. et al. *Food Res. Int.* **2019**, *122*, 230–240 [25]; [3] Wang, W. et al. *Foods* **2023**, *12*, 4207 [26]; [4] Lin, H,H. et al. this study, **2025**; [5] Chen, C. et al. *Mol. Nutr. Food Res.* **2018**, *62*, 1700483 [45]; [6] Mielgo-Ayuso, J. et al. *Br. J. Nutr.* **2014**, *111*, 1263–1271; [7] Kim, J.J. et al. *Biomed. Res. Int.* **2013**, *2013*, 920128; [8] Deng, Y.T. et al. *J. Agric. Food Chem.* **2012**, *60*, 1059–1066 [28]; [9] Gan, L. et al. *Acta. Pharmacol. Sin.* **2015**, *36*, 597–605 [48]; [10] Santamarina, A.B. et al. *PLoS One* **2015**, *10*, e0141227.	

Figure S1

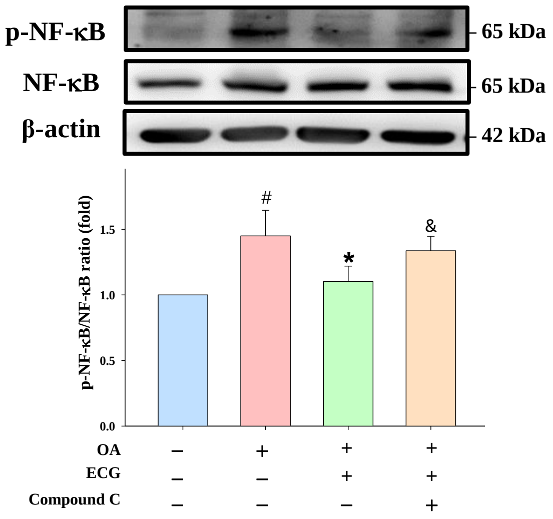

Figure S1. AMPK may be essential for the ECG-inhibited NF-κB inflammation pathway in the OA-challenged human primary hepatocytes. Hepatocytes were pre-treated with compound C (3 μM), followed by ECG (0.4 μM) and OA (0.6 mM) treatment for 24 h. Western blotting was used to assess expression of p-NF-κB and total NF-κB. β-actin was used as an internal control. Results are shown as mean ± SD ($n \geq 3$) from three independent replicate. #$p < 0.05$ vs. control; *$p < 0.05$ vs. OA group; &$p < 0.05$ vs. OA + ECG group.
